# Supplementary material for: Mapping the evolution of fertility support policies in China: A content and instrumental analysis
Source: PLoS One. 2025 Oct 9;20(10):e0332137. doi: 10.1371/journal.pone.0332137 (PMC12510515; doi:10.1371/journal.pone.0332137)
Supplement: S1 Appendix — (ZIP) [file pone.0332137.s001.zip › S1 Appendix. 226 original policy documents/219-财政部关于下达2022年积极应对人口老龄化工程和托育建设(养老、托育服务体系方向)中央基建投资预算(拨款)的通知(FBM-CLI-4-5114974).doc]

财政部关于下达2022年积极应对人口老龄化工程和托育建设(养老、托育服务体系方向)中央基建投资预算(拨款)的通知

**发布部门：** [财政部](https://www.pkulaw.com/law/chl?Aggs.IssueDepartment=60210) [机构沿革](javascript:void(0))

**发文字号：**财建〔2022〕26号

**发布日期：**2022.03.01

**实施日期：**2022.03.01

**时效性：** [现行有效](https://www.pkulaw.com/law/chl?Aggs.TimelinessDic=01)

**效力级别：** [部门工作文件](https://www.pkulaw.com/law/chl?Aggs.EffectivenessDic=XE0303)

**法规类别：** [预算管理](https://www.pkulaw.com/law/chl?Aggs.Category=03126)

财政部关于下达2022年积极应对人口老龄化工程和托育建设（养老、托育服务体系方向）中央基建投资预算（拨款）的通知
（财建〔2022〕26号）

有关省、自治区、直辖市、计划单列市财政厅（局），新疆生产建设兵团财政局：
　　根据《国家发展改革委关于下达积极应对人口老龄化工程和托育建设（养老、托育服务体系方向）2022年中央预算内投资计划的通知》（发改投资〔2022〕219号），下达你单位2022年中央基建投资（项目代码：Z135060000070）预算（拨款）指标（具体项目见附件），专项用于积极应对人口老龄化工程和托育建设（养老、托育服务体系方向）。请按规定用途安排使用，分别列入2022年支出功能分类科目和支出经济分类科目，并接受财政部当地监管局的监督。
　　为提高中央基建资金使用效益，请迅速将资金分解下达，加快预算执行进度，强化资金绩效管理与监督。按照党中央、国务院关于全面实施预算绩效管理的决策部署，请在组织预算执行中对照发改投资〔2022〕219号文件所列的绩效目标做好绩效监控，确保年度绩效目标如期实现和项目建设顺利实施。同时，请参照中央做法，会同你省（区、市、兵团）发展改革等部门及时做好绩效目标对下分解，并将有关绩效目标和指标抄送财政部当地监管局。
　　[附件1-2：](https://resources.pkulaw.cn/staticfiles/fagui/20220315/10/03/0/38c88f00e11825e9c70a347ae1e2f8d0.xls)
　　1.2022年中央基建投资预算（拨款）总表
　　2.2022年中央基建投资预算（拨款）表
　　附件3.中央基建投资绩效目标汇总表

财　政　部
　　2022年3月1日

　　附件3
　　中央基建投资绩效目标汇总表（2022年度）

| 专项名称 | | | | 积极应对人口老龄化工程和托育建设 | |
| --- | --- | --- | --- | --- | --- |
| 下达中央基建投资（万元） | | | | 740000 | |
| 总  体  目  标 | 目标1：进一步改善养老、托育服务基础设施条件，推动设施规范化、标准化建设，增强兜底保障能力，提升养老、托育服务水平。  目标2：在做好“保基本、兜底线”的基础上，继续推动增加普惠养老、普惠托育服务有效供给，逐步构建居家社区机构相协调、医养康养相结合的养老服务体系，不断发展和完善普惠托育服务体系。 | | | | |
| 绩    效    指    标 | 一级指标 | 二级指标 | 三级指标 | | 指标值 |
|  | 实施效果指标 | 产出指标 | 项目数量 | | 274个 |
|  |  |  | 建设项目验收合格率 | | ≥95% |
|  |  |  | 新建设施达到当地抗震设防要求 | | 100% |
|  |  | 效益指标 | 养老托育服务基础设施条件 | | 不断改善 |
|  | 过程管理指标 | 计划管理指标 | 投资计划分解（转发）用时 | | ≤20个工作日 |
|  |  |  | “两个责任”按项目落实到位率 | | ≥95% |
|  |  | 资金管理指标 | 中央基建投资支付率 | | ≥60% |
|  |  |  | 总投资完成率 | | ≥50% |
|  |  | 项目管理指标 | 项目开工率 | | ≥90% |
|  |  |  | 超规模、超标准、超概算项目比例 | | ≤10% |
|  |  | 监督检查指标 | 审计、督查、巡视等指出问题项目比例 | | ≤1% |

© 北大法宝：（[www.pkulaw.com](https://www.pkulaw.com/)) 专业提供法律信息、法学知识和法律软件领域各类解决方案。北大法宝为您提供丰富的参考资料，正式引用法规条文时请与标准文本核对。
欢迎查看所有[产品和服务](https://www.pkulaw.com/helps/2.html)。法宝快讯：[如何快速找到您需要的检索结果？](https://www.pkulaw.com/helps/70.html)[法宝](https://www.pkulaw.com/helps/69.html)V6有何新特色？

扫描二维码阅读原文

**原文链接：**<https://www.pkulaw.com/chl/8a73aa2a27bc5fb9bdfb.html>
